# Comprehensive analysis of prognostic tumor microenvironment-related genes in osteosarcoma patients

**DOI:** 10.1186/s12885-020-07216-2

**Published:** 2020-08-27

**Authors:** Chuan Hu, Chuan Liu, Shaoqi Tian, Yuanhe Wang, Rui Shen, Huili Rao, Jianyi Li, Xu Yang, Bo Chen, Lin Ye

**Affiliations:** 1 Department of Joint Surgery, the Affiliated Hospital of Qingdao University, Qingdao, China; 2 Department of Medical Oncology, the First Hospital of China Medical University, Shenyang, China; 3 Department of Nursing, Sir Run Run Shaw Hospital Affiliated to Zhejiang University, Hangzhou, China; 4 Wenzhou Medical University, Wenzhou, China

**Keywords:** Tumor microenvironment, Osteosarcoma, Prognosis, Immune features, Nomogram

## Abstract

**Background:**

Tumor microenvironment (TME) plays an important role in malignant tumors. Our study aimed to investigate the effect of the TME and related genes in osteosarcoma patients.

**Methods:**

Gene expression profiles and clinical data of osteosarcoma patients were downloaded from the TARGET dataset. ESTIMATE algorithm was used to quantify the immune score. Then, the association between immune score and prognosis was studied. Afterward, a differential analysis was performed based on the high- and low-immune scores to determine TME-related genes. Additionally, Cox analyses were performed to construct two prognostic signatures for overall survival (OS) and disease-free survival (DFS), respectively. Two datasets obtained from the GEO database were used to validate signatures.

**Results:**

Eighty-five patients were included in our research. The survival analysis indicated that patients with higher immune score have a favorable OS and DFS. Moreover, 769 genes were determined as TME-related genes. The unsupervised clustering analysis revealed two clusters were significantly related to immune score and T cells CD4 memory fraction. In addition, two signatures were generated based on three and two TME-related genes, respectively. Both two signatures can significantly divide patients into low- and high-risk groups and were validated in two GEO datasets. Afterward, the risk score and metastatic status were identified as independent prognostic factors for both OS and DFS and two nomograms were generated. The C-indexes of OS nomogram and DFS nomogram were 0.791 and 0.711, respectively.

**Conclusion:**

TME was associated with the prognosis of osteosarcoma patients. Prognostic models based on TME-related genes can effectively predict OS and DFS of osteosarcoma patients.

## Background

Osteosarcoma is the most common bone tumor, especially in children and adolescents [1]. It was reported that approximately 60% of patients are between 10 and 20 years old and osteosarcoma is considered as the second leading cause of death in this age group [2]. Currently, surgery and chemotherapy are still major treatments for osteosarcoma patients, and these therapies are constantly improving in recent years. However, due to the susceptibility of local aggressiveness and lung metastasis in osteosarcoma patients, the prognosis of osteosarcoma remains unfavorable [3]. Previous studies indicated that the 5-years survival rates were 27.4 and 70% in metastatic and non-metastatic patients, respectively [4]. Therefore, it is necessary to investigate the mechanism of pathogenesis and progression of osteosarcoma and accurately classify the risk of patients.

Recently, an increasing number of diagnostic and prognostic biomarkers of osteosarcoma patients have been identified. For example, Chen et al. [5] reported that tumor suppressor p27 is a novel biomarker for the metastasis and survival status in osteosarcoma patients. Moreover, Huang et al. [6] discovered that dysregulated circRNAs serve as prognostic and diagnostic biomarkers in osteosarcoma patients, and the relative potential mechanism mainly attributes to the regulation of downstream signaling pathways by sponging microRNA. In addition, lncRNA [7], microRNA [8], and many clinical data [9] were also identified as prognostic biomarkers for osteosarcoma patients. However, osteosarcoma is one of the malignant cancers entities characterized by the high level of heterogeneity in humans. Therefore, it is necessary to find accurate biomarkers for osteosarcoma.

In recent years, researchers have paid more and more attention to the role of the tumor microenvironment (TME) in malignant tumors. The function of TME in the tumorigenesis, progression, and therapy of tumors have been initially understood [10, 11]. More importantly, Estimation of STromal and Immune cells in MAlignant Tumor tissues using Expression data (ESTIMATE), an algorithm to quantify the score of immune cells and stromal cells by analyzing the gene expression data, was developed in 2013 [12]. Based on the algorithm, the prognostic value of immune and stromal cells in bladder cancer, acute myeloid leukemia, gastric cancer, cervical squamous cell carcinoma, adrenocortical carcinoma, clear cell renal cell carcinoma, hepatocellular carcinoma, thyroid cancer, and cutaneous melanoma have been reported [13–23]. Generally, the above research indicated that TME can serve as the prognostic biomarker in tumors, and many TME-related genes were determined as the prognostic genes. However, the role of TME and TME-related genes in osteosarcoma patients remains unclear.

In the present study, gene expression data and corresponding clinicopathologic data were obtained from The Therapeutically Applicable Research to Generate Effective Treatments (TARGET) dataset. Then, the ESTIMATE algorithm was performed to quantify the immune score of osteosarcoma and the TME-related genes were identified by the differential expression analysis. Subsequently, the prognostic value of TME and TME-related genes were determined by a series of bioinformatics methods.

## Methods

### Gene expression datasets

Level 3 data of gene expression profiles and corresponding clinical data of osteosarcoma patients were downloaded from TARGET dataset (https://ocg.cancer.gov/programs/target, accessed on Oct 11, 2019). The corresponding clinicopathologic data included in the present study were age, gender, race, ethnicity, tumor site, and metastatic status. After data were extracted from the public domain, the ESTIMATE, an algorithm inferring tumor purity, stromal score, and immune cell admixture from expression data, was performed to evaluate the immune score by using the estimate package in R software (version 3.6.1) [12]. Meanwhile, the messenger RNA(mRNA) expression profiles and clinical data of two cohorts, including GSE21257 [24] and GSE39055 [25], were obtained from the Gene Expression Omnibus as external validation cohorts.

### Survival analysis and correlation analysis

After scores were obtained, patients were divided into high-score group and low-score group according to the median of the immune score. The Kaplan-Meier survival analysis with log-rank test was performed to estimate the differences of overall survival (OS) and disease-free survival (DFS) between high- and low-score cohorts. In addition, the association between clinicopathologic data and TME score was also studied. Mann-Whitney signed-rank test was performed to compare the differences of immune score between each clinical group. All statistical analyses in the present study were performed using R software. Except for the special instructions, *p* value< 0.05 (two-side) was identified as statistically significant in the present study.

### Differentially expressed gene analysis

Differentially expressed gene (DEG) analysis was performed by comparing the protein-coding genes expression between the low-immune score group and the high-immune score group. The limma package in R software was used to perform the differential analysis and genes with |log FC| > 1.0 and adjusted *p*-value (q value) < 0.05 were identified as DEGs [26].

To further understand the function of DEGs identified in the present study, Gene Ontology (GO), including biological processes (BP), molecular functions (MF), and cellular components(CC) and Kyoto Encyclopedia of Genes and Genomes (KEGG) analysis were performed by clusterProfiler package in R software [27].

### Evaluation of association with immune cells

To further investigate the association between DEGs and immune cells, the CIBERSORT package was used to estimate the relative proportions of 22 types of immune cells [28]. Meanwhile, the “ConsensusClusterPlus” package was used to cluster in an unbiased and unsupervised manner based on the overlapping DEGs [29]. Cumulative distribution function (CDF) and relative change in area under the CDF curve were used to determine the optimal number of clusters k. Then, Mann-Whitney signed-rank test was performed to study the difference of immune cells proportion between the clusters and the violin plot was established to show the differences of immune cells among clusters [30].

### Survival analysis of DEGs

Based on the DEGs, the univariate COX analysis was performed to determine the prognostic value of immune-related genes. Then, the OS-related genes were validated in the GSE21257 dataset, while the DFS-related genes were validated in the GSE39055 dataset. Only genes successfully validated were selected for further analysis. Afterward, based on the validated genes, the multivariate COX analysis was performed to establish the prognostic signature for predicting the prognosis of osteosarcoma patients. The risk score for each patient was calculated based on the coefficient from the multivariate COX analysis and the corresponding gene expression. Meanwhile, all patients were divided into the high- and low-risk groups according to the median of the risk score. The survival receiver operating characteristic (ROC) curve was used to show the discrimination of signatures, and the Kaplan-Meier survival curve with the log-rank test was generated to show the differences of OS and DFS between high- and low-risk groups. In addition, the risk score of patients in the validation cohort was also calculated according to the aforementioned risk signature. The Kaplan-Meier survival curve and survival ROC curve were generated to show the predictive ability of the signature in the validation cohort.

### Development of a nomogram for osteosarcoma patients

Nomogram is a tool to visualize the predictive model and convenient for clinical practice. Therefore, we attempted to develop a nomogram based on the TME-related genes signature and clinicopathologic data to predict the prognosis of osteosarcoma patients. Firstly, the univariate COX analysis was performed to filter prognostic variables, which will be further included in the multivariate COX analysis. Secondly, based on independent prognostic variables, two nomograms were established for predicting the OS and DFS, respectively. The C-index was used to assess the discriminatory performance of the nomogram, which range from 0.5 to 1 [31]. A C-index of 0.5 means agreement by chance and a C-index of 1 represents perfect discriminatory performance. The higher value of the C-index, the better performance of the nomogram is. Furthermore, the calibration curves of 1-, 2-, and 3-year were developed to evaluate the effectiveness of nomograms.

## Results

### Immune significantly associated with the prognosis of osteosarcoma patients

**Fig. 1:**
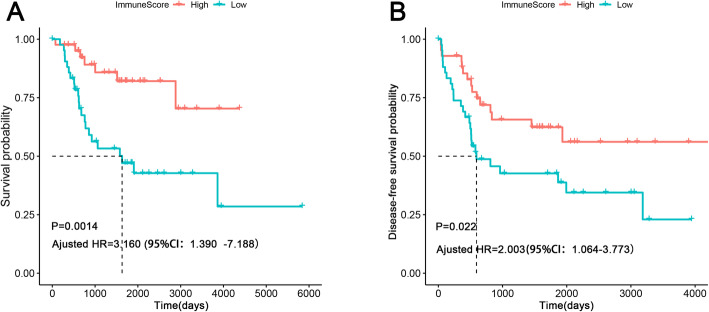
Association between immune score and prognosis in osteosarcoma patients. **a** Kaplan-Meier survival analysis of overall survival for patients with high vs. low immune score; **b** Kaplan-Meier survival analysis of disease-free survival for patients with high vs. low immune score

85 osteosarcoma patients were included in the present study, including 48 males and 37 females. The immune score of the cohort range from − 1459.56 to 2581.96. To study the relationship between the immune score and the prognosis of osteosarcoma patients, 42 patients were incorporated into the low-immune score group, while the remaining 43 patients were incorporated into the high-immune score group. The survival analysis indicated that patients with higher immune score had a favorable OS and DFS (Fig. 1a and b). After adjusted age, tumor site, and metastatic status, the immune score still was a prognostic variable for both OS and DFS(Fig. 1a and b). In addition, the relationship between immune score and clinical features was also investigated. However, there was no significant relationship between immune score and clinical variables (Supplementary Figure 1A-1C).

### Differential expression analysis

**Fig. 2:**
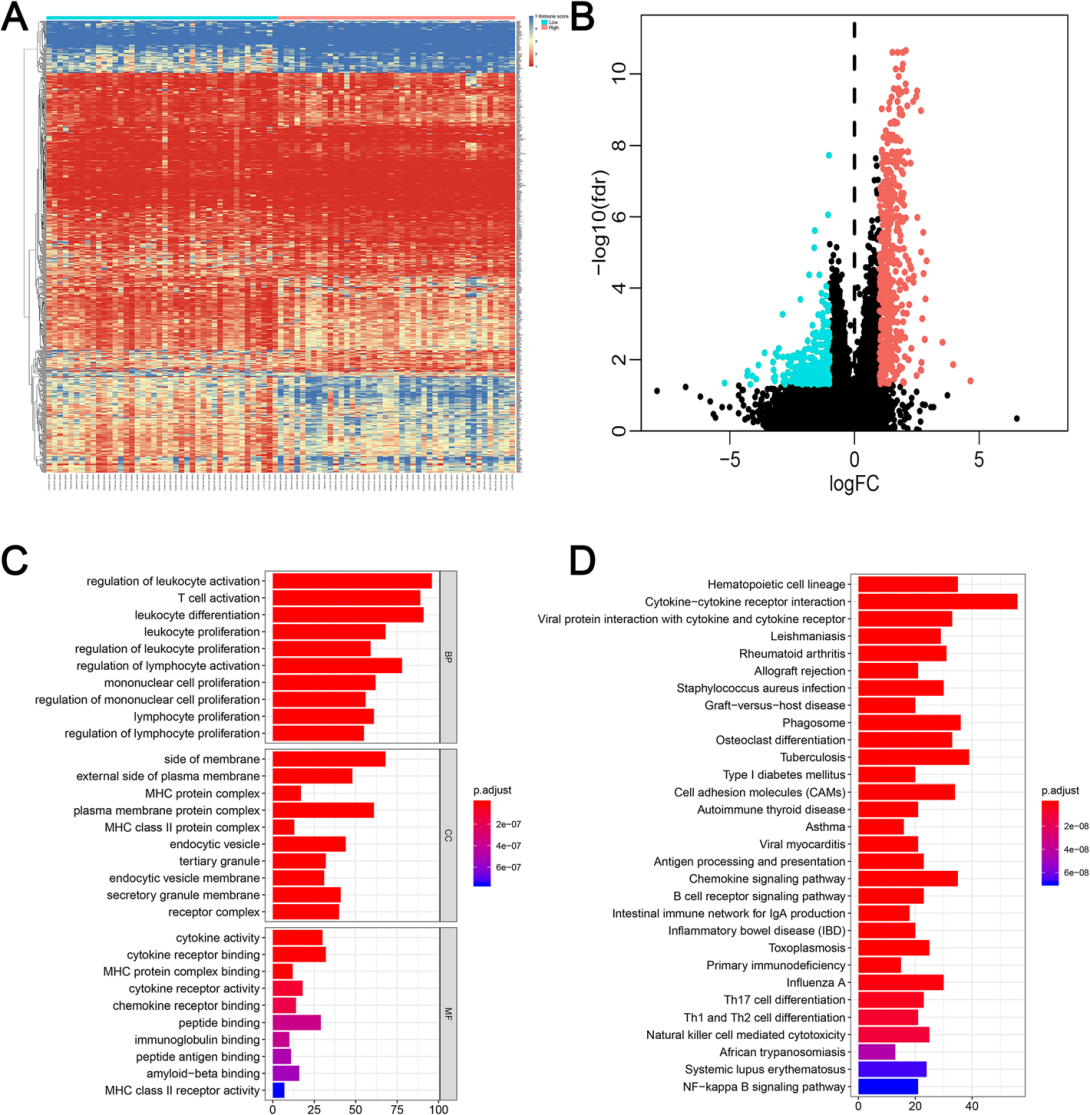
Differentially expressed genes with the immune score in osteosarcoma patients. **a** Heatmap of significantly differentially expressed genes based on immune score; **b** The volcano figure to show the upregulated and downregulated genes. **c** GO analysis of differentially expressed genes. **d** KEGG of differentially expressed genes. GO: Gene Ontology; KEGG: Kyoto Encyclopedia of Genes and Genomes

According to the median of the immune score, 85 patients were divided into high-score (*n* = 43) and low-score group (*n* = 42). There were 769 differentially expressed genes between two groups, which include 498 upregulated genes and 271 downregulated genes (Fig. 2a, b, and Supplementary Table 1). To further understand the function of 769 DEGs, GO analysis and KEGG analysis were performed. The top 10 significant results of GO analysis among three types were illustrated in Fig. 2c. Interestingly, we can find that the results of GO analysis are mostly associated with immunity, which further verify that the immune-related DEGs are associated with immune features. In addition, the results of KEGG also confirmed it. Such as “Phagosome”, “Autoimmune thyroid disease”, “Antigen processing and presentation”, “B cell receptor signaling pathway”, “Intestinal immune network for IgA production”, “Inflammatory bowel disease”, “Primary immunodeficiency”, “Th1 and Th2 cell differentiation”, “Th17 cell differentiation”, “Natural killer cell mediated cytotoxicity”, and “NF−kappa B signaling pathway” (Fig. 2d).

### Evaluation of DEGs and immune cells

**Fig. 3:**
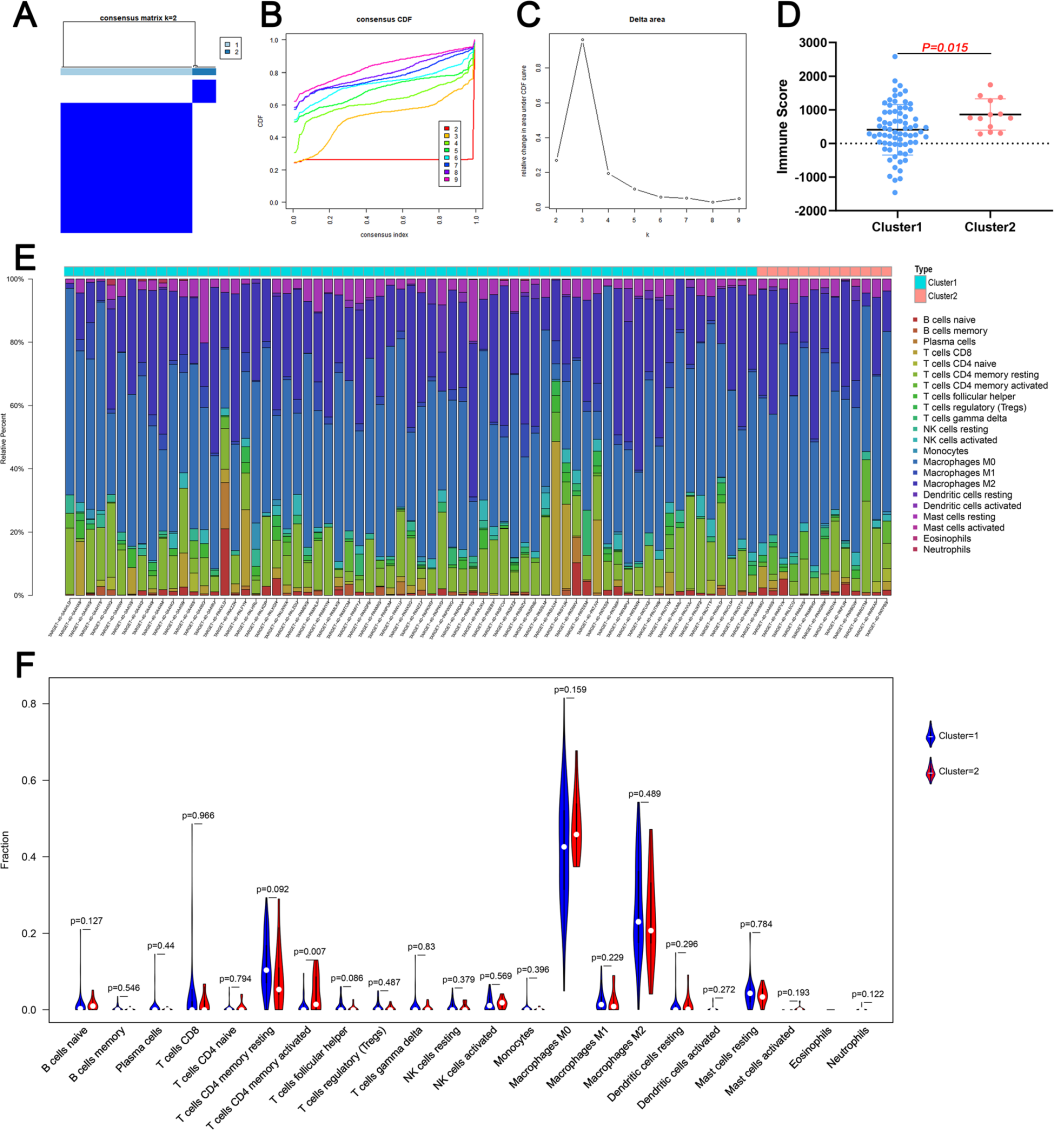
The immune landscape of the tumor microenvironment. **a**-**c** Unsupervised clustering of all samples based on the overlapping DEGs; **d** Comparison of immune score between two clusters; **e** The distribution of 22 types of immune cells in osteosarcoma patients; **f** The comparison of 22 types of immune cells between clusters. DEG: Differentially expressed gene

To further understand the molecular heterogeneity of osteosarcoma, unsupervised consensus analysis was performed to divide patients into subgroups to explore whether immune-related genes presented discernable patterns. Based on the consensus matrix heat map, patients were clearly divided into two clusters(Fig. 3a). In addition, by comprehensively analyzing the relative change in area under the cumulative distribution function, two clusters were determined (Fig. 3b-c). The immune score between two clusters was significantly different (Fig. 3d). In addition, the proportion of 22 types of immune cells in osteosarcoma patients was illustrated in a barplot (Fig. 3e). Interestingly, we can see that the T cells CD4 memory activated of cluster 2 is significantly higher than cluster 1 (Fig. 5f).

### Prognostic value of TME-related genes

**Fig. 4:**
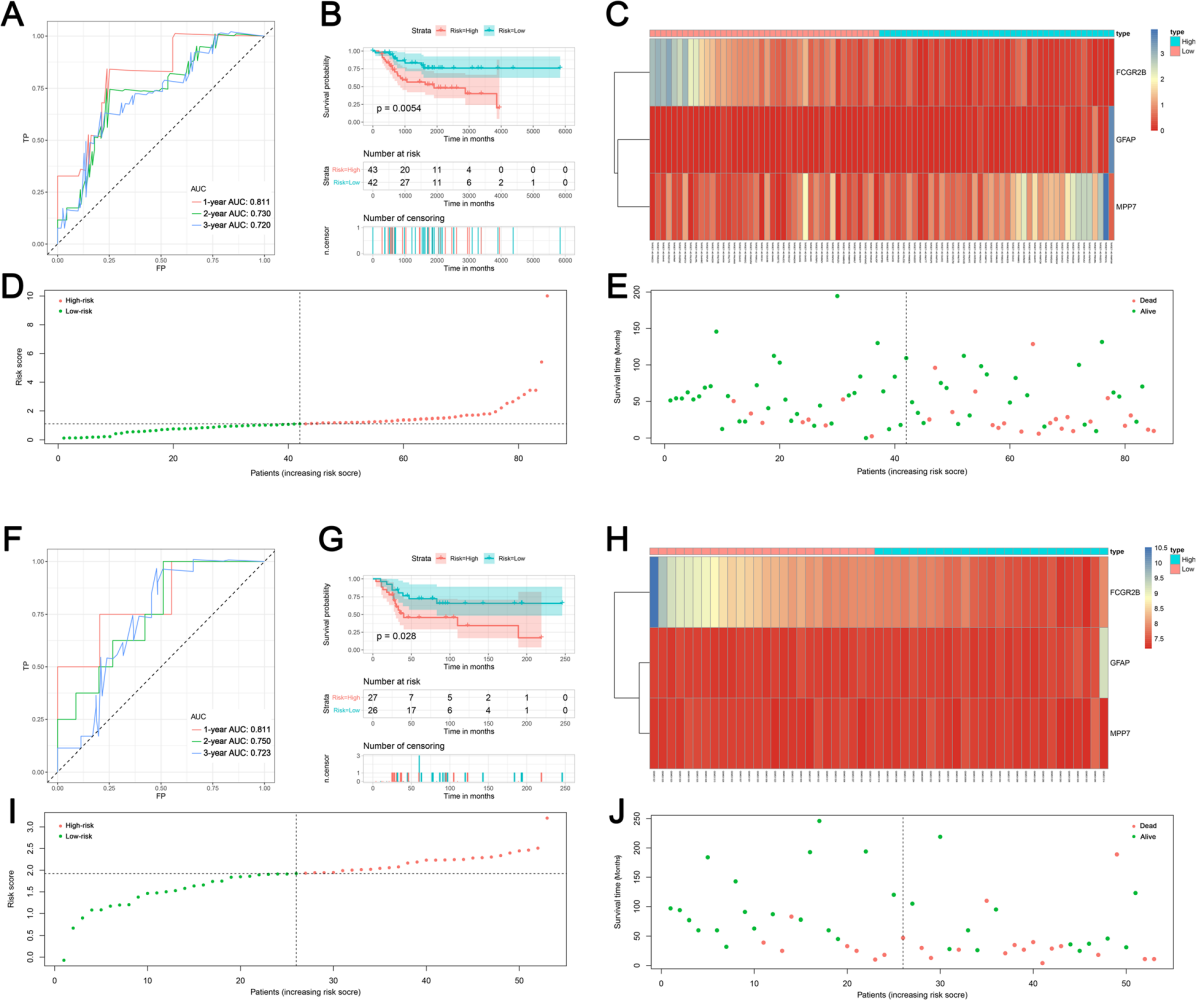
Establishment and validation of the prognostic model for overall survival based on significant DEGs; **a** Receiver operating characteristic curves of prognostic signature in the training cohort; **b** The survival curve showed the different overall survival status between high- and low-risk patients. **c** The heat map showed the expression of prognostic genes in the training cohort. **d** The risk curve of each sample reordered by risk score; **e** The scatter plot showed the overall survival status of osteosarcoma patients in the training cohort; **f** Receiver operating characteristic curves of prognostic signature in validation cohort; **g** The survival curve showed the different overall survival status between high- and low-risk patients. **h** The heat map showed the expression of prognostic genes in the validation cohort. **i** The risk curve of each sample reordered by risk score; **j** The scatter plot showed the overall survival status of osteosarcoma patients in the validation cohort

**Fig. 5:**
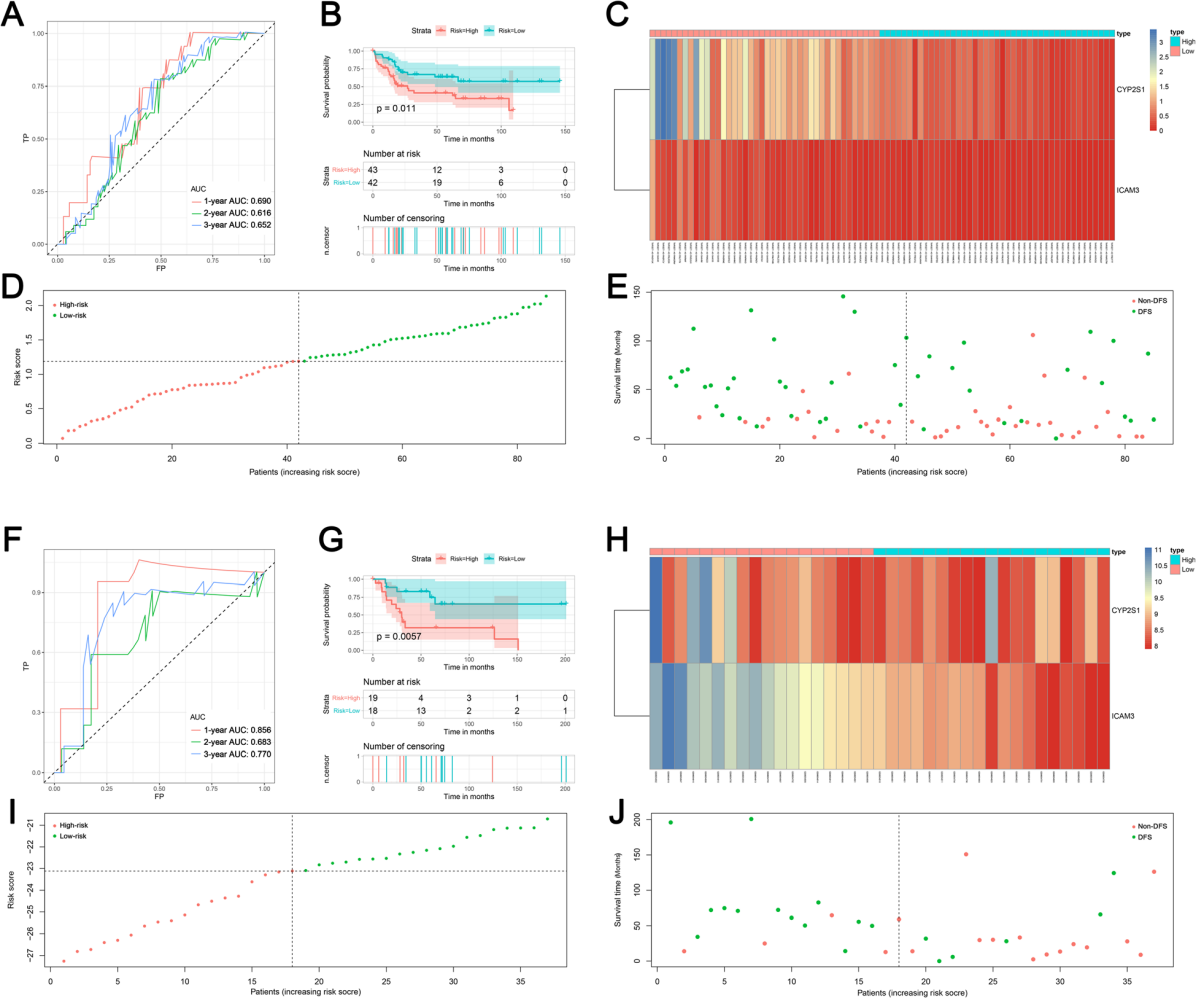
Establishment and validation of the prognostic model for disease-free survival based on significant DEGs; **a** Receiver operating characteristic curves of prognostic signature in the training cohort; **b** The survival curve showed the different disease-free status between high- and low-risk patients. **c** The heat map showed the expression of prognostic genes in the training cohort. **d** The risk curve of each sample reordered by risk score; **e** The scatter plot showed the disease-free status of osteosarcoma patients in the training cohort; **f** Receiver operating characteristic curves of prognostic signature in validation cohort; **g** The survival curve showed the different disease-free status between high- and low-risk patients. **h** The heat map showed the expression of prognostic genes in the validation cohort. **i** The risk curve of each sample reordered by risk score; **j** The scatter plot showed the disease-free status of osteosarcoma patients in the validation cohort

Previous studies indicated that TME-related genes can serve as the prognostic biomarker for tumor patients. Hence, we performed the univariate COX analysis to identify prognostic DEGs. The results showed that 160 and 120 genes were identified as OS- and DFS-related DEGs, respectively (Supplementary Table 2 and 3). Afterward, five OS-related genes were successfully validated in the GSE21257 data set, and five DFS-related genes were successfully validated in the GSE39055 cohort. Furthermore, multivariate COX analysis was performed and two prognostic signatures were generated for predicting the OS and DFS, respectively. The risk score for predicting the OS was as follows: risk score = FCGR2B*-0.766 + GFAP*0.702 + MPP7*0.387. In addition, the risk score for predicting the DFS was as follows: risk score = CYP2S1*-0.574 + ICAM3*-2.015. The AUC values of OS-related signature were 0.811, 0.730, and 0.720 in 1-, 2-, and 3-year, respectively (Fig. 4a), and the AUC values of DFS-related signature were 0.690, 0.616, and 0.652 in 1-, 2-, and 3-year, respectively (Fig. 5a). Moreover, survival curves showed that patients in the high-risk group had worse OS and DFS compared with the low-risk patients (Figs. 4b and 5b). Heat maps, risk score plots, and survival status were generated to show the distinction between high-risk patients and low-risk patients (Figs. 4c-e and 5c-e). Then, both signatures were validated in independent cohorts. For OS signature, the AUC values of validation cohort were 0.811, 0.750, and 0.723 at 1-, 2-, and 3-year (Fig. 4f). For DFS signature, the AUC values of validation cohort were 0.856, 0.683, and 0.770 at 1-, 2-, and 3-year (Fig. 5f). Additionally, in both validation cohorts, survival curves showed that low-risk patients were favorable prognosis than high-risk patients (Figs. 4g and 5g). Heat maps, risk score plots, and survival status of validation cohorts were also generated to show the distinction between high-risk patients and low-risk patients (Figs. 4h-j and f 5h-j).

### Development of a nomogram for osteosarcoma patients

**Fig. 6:**
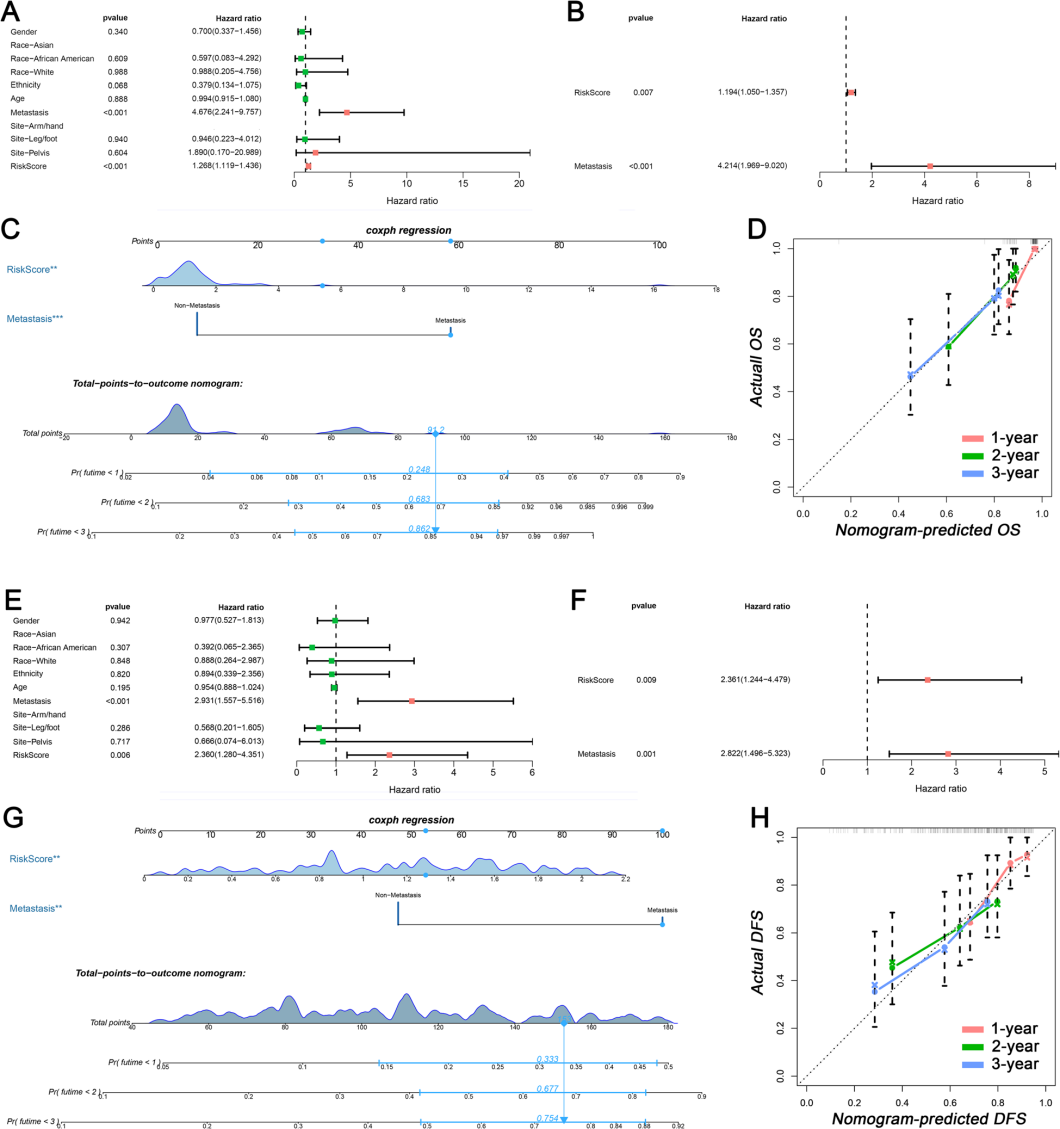
Nomograms based on the tumor microenvironment related genes for osteosarcoma patients. **a** Univariate COX analysis of overall survival-related variables; **b** Multivariate COX analysis of overall survival-related variables; **c** Nomogram for predicting the overall survival in osteosarcoma patients; **d**1-, 2-, and 3-year calibration curveS of overall survival nomogram; **e** Univariate COX analysis of disease-free survival-related variables; **f** Multivariate COX analysis of disease-free survival-related variables; **g** Nomogram for predicting the disease-free survival in osteosarcoma patients; **h**1-, 2-, and 3-year calibration curveS of disease-free survival nomogram

To generate a nomogram for clinical use, the COX analysis was performed to select the clinical prognostic variables. In the univariate COX analysis, risk score and metastatic status were identified as both OS- and DFS-related variables (Fig. 6a and e). Afterward, risk score and metastatic status were determined as both independent OS- and DFS-related variables in the multivariate COX analysis (Fig. 6b and f). Based on independent variables, two nomograms were established for predicting the OS and DFS in osteosarcoma patients, respectively (Fig. 6c and g). The C-index values were 0.739 and 0.687 in OS nomogram and DFS nomogram, respectively. The results of C-index mean that both two nomograms have good discrimination. Meanwhile, to evaluate the calibration of nomograms, six calibration curves were generated and the results showed that the predictive curves were close to the ideal curve (Fig. 6d and h), which indicated a good calibration.

## Discussion

The relationship between TME and tumor have been widely studied in recent years. In the present study, ESTIMATE algorithm was utilized to quantify the immune score based on gene expression profiles in 85 osteosarcoma patients from TARGET database. We confirmed that the TME is significantly associated with the prognosis of osteosarcoma patients, including OS and DFS. In addition, functional enrichment analyses of TME-related genes indicated that immune-related processes known to contribute to tumor progression. More importantly, DEGs based on the TME were identified as important prognostic biomarkers for osteosarcoma patients, and two nomograms were developed for predicting the OS and DFS of osteosarcoma patients, respectively.

In recent years, an increasing number of studies focused on the carcinogenesis and progression of tumors based on the TME, and the ESTIMATE algorithm is one of the most important quantitative tools for this research field. Based on the ESTIMATE algorithm, the association between the prognosis and TME has been initially elucidated in some tumors, such as cervical squamous cell carcinoma, gastric cancer, cutaneous melanoma, acute myeloid leukemia, bladder cancer, and clear cell renal carcinoma [13, 16, 17, 19–23]. However, previous studies indicated that TME scores serve as a different role in different tumors. For example, for hepatocellular carcinoma, gastric cancer, acute myeloid leukemia, bladder cancer, and clear cell renal carcinoma, patients with high immune score have a worse prognosis [14, 16, 17, 20–23]. However, for cervical squamous cell carcinoma, adrenocortical carcinoma, and cutaneous melanoma, patients with high immune score have a favorable prognosis [13, 18, 19]. Therefore, we can find great heterogeneity among different tumors from the perspective of TME. For osteosarcoma patients, the present study indicated that patients with higher immune score had a better OS and DFS. Hence, the present study indicated that immune cells infiltrating tumor tissue may play an important role in suppressing tumor progression.

In our research, 769 TME-related genes were identified by comparing the high-score and low-score osteosarcoma patients. The functional enrichment, including GO and KEGG analyses, showed that TME-related genes were mainly involved in the immune features, such as regulation of leukocyte activation, MHC protein complex, MHC protein, and complex binding. More importantly, the unsupervised cluster analysis based on DEGs was performed and all patients were divided into two clusters. Immune score and T cell CD4 memory activated fraction were significant difference between two clusters, which further elucidated the relationship between DEGs and immune features.

Due to the poor prognosis of osteosarcoma patients, identifying robust prognostic biomarker is very important. The tumor immune microenvironment is closely related to the prognosis of bone tumor patients. Emilie et.al [24] performed the first genome-wide study to describe the role of immune cells in osteosarcoma and found that tumor-associated macrophages are associated with reduced metastasis and improved survival in high-grade osteosarcoma. Recently, the prognostic signature based on TME-related genes have been established for many tumors [18, 20, 32], but only one study focused on osteosarcoma patients [33]. Compared with the study performed by Zhang et al. [33], we think that our research have some advantages. Firstly, our signatures were established based on several validated genes, and both two signatures were successfully validated in independent cohorts. Secondly, the outcome of DFS was not reported in the previous study. As reported in published studies, tumor recurrence is a terrible medical problem for osteosarcoma patients, and the 5-year survival rate for osteosarcoma patients with metastasis or relapse remains disappointing [34, 35]. Hence, the DFS nomogram can improve the management of osteosarcoma patients. Finally, two nomograms incorporated TME-related signature and clinical variables were established in our research, which further facilitated the clinical application of our findings.

In our research, five genes were incorporated into the final prognostic signatures. FCGR2B, GFAP, and MPP7 were identified and validated as OS-related biomarkers, while CYP2S1 and ICAM3 were DFS-related biomarkers. The role of these genes in tumor prognosis had been widely reported in previous studies [36–40]. FCGR2B has been confirmed as an immune-related gene previously [41]. Although the relationship between FCGR2B and prognosis in sarcoma patients had not been reported, the prognostic value of FCGR2B had been widely confirmed in other cancers, such as hepatocellular carcinoma and glioblastoma [36, 42]. In addition, New M et.al [37] demonstrated that MPP7 is novel regulators of autophagy, which was thought to be responsible for the prognosis of pancreatic ductal adenocarcinoma. CYP2S1, described as Cytochrome P450 Family 2 Subfamily S Member 1, was reported significantly associated with colorectal cancer. In primary colorectal cancer, CYP2S1 was present at a significantly higher level of intensity compared with normal colon [43]. More importantly, the presence of strong CYP2S1 immunoreactivity was associated with poor prognosis [43]. The role of ICAM3 in cancer was also widely reported in published studies, and the Akt pathway plays an important role in the impact of ICAM3 on tumors. YG Kim et.al [44] reported that ICAM3 can induce the proliferation of cancer cells through the PI3K/Akt pathway. Additionally, JK Park et.al showed that the ICAM3 can enhance the migratory and invasive potential of human non-small cell lung cancer cells by inducing MMP-2 and MMP-9 via Akt pathway [45] showed that the ICAM3 can enhance the migratory and invasive potential of human non-small cell lung cancer cells by inducing MMP-2 and MMP-9 via Akt pathway.

Although the role of TME and TME-related genes in osteosarcoma patients have been initially studied by bioinformatic and statistical analyses in our research, some limitations should be elucidated. Firstly, the treatment information cannot be obtained from the TARGET database, which may influence the prognosis of osteosarcoma patients. Secondly, two nomograms were generated and showed good performance in our study. However, external validation by a large cohort is needed. Thirdly, many independent prognostic genes for osteosarcoma patients were identified in the present study, but the potential mechanism to influence osteosarcoma remains unclear. Finally, in the training cohort, 160 and 120 DEGs were identified as OS- and DFS-related DEGs, respectively. However, only five OS- and five DFS-related genes were identified in the validation cohort. The different age structures, smaller sample sizes and the platform covering only part of the genes may contribute to this result.

## Conclusion

In conclusion, TME plays an important role in osteosarcoma patients and related with the progression of the tumor. Moreover, TME-related genes can serve as prognostic biomarkers in osteosarcoma patients. However, further researches are needed to study the potential mechanism and validate the nomogram that developed in our present study.

## Supplementary information

**Additional file 1.**

**Additional file 2.**

**Additional file 3.**

**Additional file 4.**

## Data Availability

The data of this study are from TARGET and GEO database.

## Abbreviations

TME: Tumor microenvironment
DEG: Differentially expressed genes
OS: Overall survival
DFS: Diseases-free survival
ROC: Receiver characteristic curve
ESTIMATE: Estimation of STromal and Immune cells in MAlignant Tumor tissues using Expression data
TARGET: Therapeutically Applicable Research to Generate Effective Treatments
GO: Gene Ontology
BP: Biological processes
MF: Molecular functions
CC: Cellular components
KEGG: Kyoto Encyclopedia of Genes and Genomes
CDF: Cumulative distribution function
