# Supplementary figures and images for: Comprehensive analysis of prognostic tumor microenvironment-related genes in osteosarcoma patients
Source: BMC Cancer. 2020 Aug 27;20:814. doi: 10.1186/s12885-020-07216-2 (PMC7450807; doi:10.1186/s12885-020-07216-2)

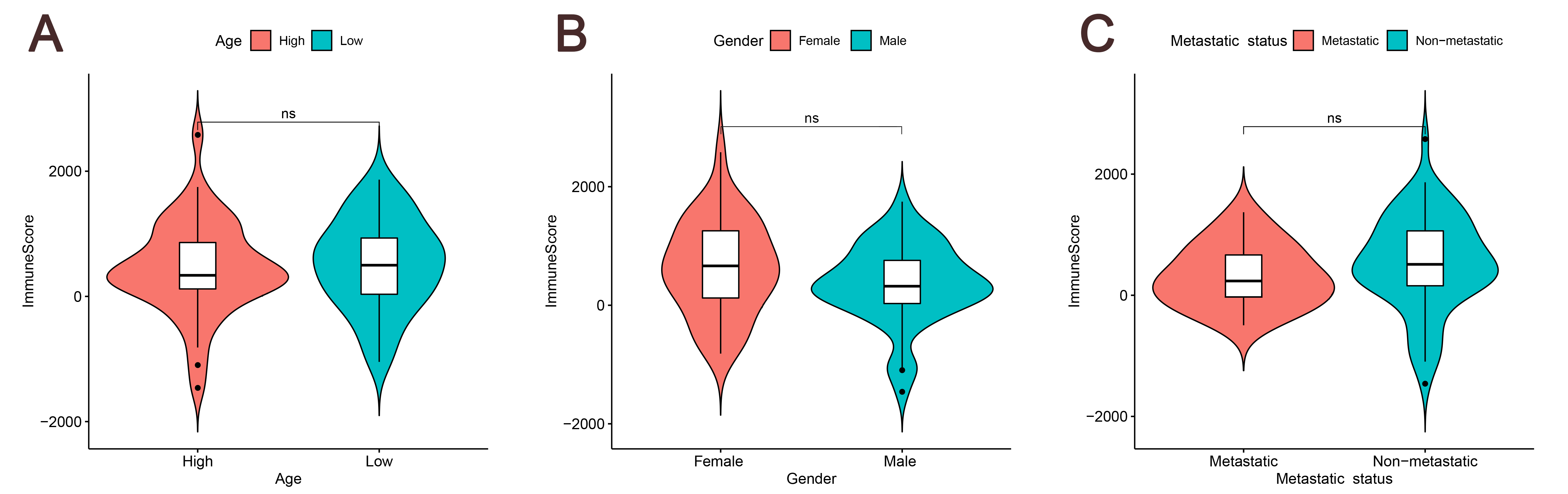

Supplement: Supplementary file 1 — Additional file 1. [file 12885_2020_7216_MOESM1_ESM.tif]
